# Serum Metabolomics Reveals Carnitine Metabolism as a Possible Central Metabolic Axis of Pemafibrate Action

**DOI:** 10.3390/ijms27146252

**Published:** 2026-07-14

**Authors:** Chufang Qian, Xuguang Zhang, Zhe Zhang, Kazuhiro Tanabe, Chihiro Hayashi, Hiroki Kittaka, Qianqian Zheng, Jiali Chen, Sakura Yuki, Xiyue Yang, Yifan Diao, Takero Nakajima, Takanobu Iwadare, Takefumi Kimura, Makoto Nakamuta, Naoki Tanaka

**Affiliations:** 1 Department of Global Medical Research Promotion, Shinshu University Graduate School of Medicine, Matsumoto 390-8621, Japan; 22hm182j@shinshu-u.ac.jp (C.Q.); xgzhang.cj@gmail.com (X.Z.); zhangzhezz0914@163.com (Z.Z.); 19470008@hebmu.edu.cn (Q.Z.); 23hm122j@shinshu-u.ac.jp (J.C.); 24hm134f@shinshu-u.ac.jp (S.Y.); 24hm185a@shinshu-u.ac.jp (Y.D.); nakat@shinshu-u.ac.jp (T.N.); 2 Department of Clinical Laboratory, The Second Hospital of Hebei Medical University, Shijiazhuang 050000, China; 3 Postdoctoral Mobile Station of Clinical Medicine, Hebei Medical University, Shijiazhuang 050017, China; 4 Department of Gastroenterology, University-Town Hospital, The Fourth Clinical College of Chongqing Medical University, Chongqing 401331, China; 5 Medical Solution Promotion Department, Medical Solution Segment, LSI Medience Corporation, Tokyo 174-8555, Japan; kazuhirotanabe77@gmail.com (K.T.); hayashi.chihiro@ma.medience.co.jp (C.H.); 6 Kyushu Professional Search Limited Liability Partnership, Fukuoka 819-0388, Japan; kittaka.hiroki@ma.medience.co.jp; 7 Department of Traditional and Western Medical Hepatology, Hebei Medical University Third Hospital, Shijiazhuang 050061, China; 8 Department of Gastroenterology, Shinshu University School of Medicine, Matsumoto 390-8621, Japan; iwnb.0522@gmail.com (T.I.); kimuratakefumii@yahoo.co.jp (T.K.); 9 Institute for Biomedical Sciences, Shinshu University, Matsumoto 390-8621, Japan; 10 Department of Gastroenterology, Kyushu Medical Center, Fukuoka 810-8563, Japan; mako330711naka@gmail.com; 11 International Relations Office, Shinshu University School of Medicine, Matsumoto 390-8621, Japan

**Keywords:** pemafibrate, selective peroxisome proliferator-activated receptor α modulator, carnitine, carnitine o-acetyltransferase, carnitine O-octanoyltransferase, metabolomics

## Abstract

Pemafibrate (PEM), a novel selective peroxisome proliferator-activated receptor α modulator, is widely used to treat dyslipidemia, yet its systemic metabolic effects remain incompletely defined. We performed untargeted serum metabolomics in patients with hypertriglyceridemia at baseline and at 2 and 8 weeks after PEM treatment. PEM increased cystine, L-methionine, uridine, and L-carnitine, while decreasing lipid-related metabolites, including lysophosphatidylcholines, adenosine, and erucic acid (22:1). Circulating carnitine levels rose progressively, with a significant elevation at 8 weeks, whereas ketone bodies showed only modest, non-significant increases. Consistently, increases in circulating and tissue carnitine were also observed in male 8-week-old C57BL/6J mice treated with a clinically relevant dose of PEM for 2 weeks. Mechanistically, PEM did not significantly alter genes involved in carnitine biosynthesis or transport, but upregulated hepatic carnitine O-acetyltransferase and carnitine O-octanoyltransferase, key enzymes of carnitine utilization and turnover. Collectively, these findings suggest that enhanced carnitine metabolism represents an important metabolic axis of PEM action.

## 1. Introduction

Pemafibrate (PEM) is a novel selective peroxisome proliferator-activated receptor α (PPARα) modulator (SPPARMα) and is widely used for the treatment of dyslipidemia [1]. PPARα is involved not only in lipid metabolism but also in the regulation of energy homeostasis and mitochondrial function, and its activation can elicit diverse metabolic responses across different tissues [2,3,4,5]. Previous studies have shown that pharmacological activation of PPARα induces the expression of genes involved in fatty acid uptake and β-oxidation, thereby improving lipid metabolic status [2]. However, because PPARα regulates multiple metabolic pathways, it remains unclear whether PEM induces systemic metabolic changes in humans beyond its lipid-lowering effects.

To evaluate systemic metabolic changes associated with pharmacological intervention, approaches capable of broadly assessing circulating metabolites are required. Metabolomics enables the comprehensive analysis of low-molecular-weight metabolites and has been widely applied to characterize systemic metabolic responses to physiological and pharmacological interventions [6,7]. In particular, untargeted liquid chromatography-mass spectrometry-based metabolomic analysis allows the detection of metabolic changes without prior assumptions regarding specific pathways and has been used to investigate drug-induced metabolic alterations *in vivo* [7,8]. Therefore, in the present study, we performed untargeted serum metabolomics in patients with hypertriglyceridemia before and after PEM treatment to assess PEM-induced metabolic changes. In addition, we further investigated the molecular mechanism by which PEM alters carnitine metabolism using mice treated with a clinically relevant dose of PEM [9].

## 2. Results

### 2.1. Serum Metabolomics Uncovers Altered Carnitine Metabolism After PEM Treatment in Patients with Hypertriglyceridemia

We obtained serum samples from 7 patients with hypertriglyceridemia at the initiation of PEM treatment (0 W) and 2 weeks (2 W) and 8 weeks (8 W) after the treatment. Serum samples were collected after overnight fasting. The baseline characteristics of the seven patients, including age, sex, underlying diseases, and lipid profiles, are summarized in Supplementary Table S1. Indeed, serum triglyceride levels were significantly reduced after the treatment, whereas total cholesterol, high-density-lipoprotein cholesterol, and low-density-lipoprotein cholesterol showed relatively modest changes (Supplementary Figure S1).

To investigate the effects of PEM on whole-body metabolism, untargeted metabolomic profiling was performed on human serum samples collected at baseline (0 W), week 2 (2 W), and week 8 (8 W). At week 2, volcano plot analysis identified ten metabolites that were significantly altered compared with baseline by paired two-tailed Student’s *t*-test (Supplementary Figure S2). Among them, 5-methylcytidine and cystine were increased, whereas lactic acid, pyruvate, citrate, β-hydroxypyruvic acid, ergothioneine, D-glucarate, adrenosterone, and lysophosphatidylcholine (LPC) 17:0 were decreased (Supplementary Figures S1–S3 and Supplementary Tables S2 and S3).

**Figure 1:**
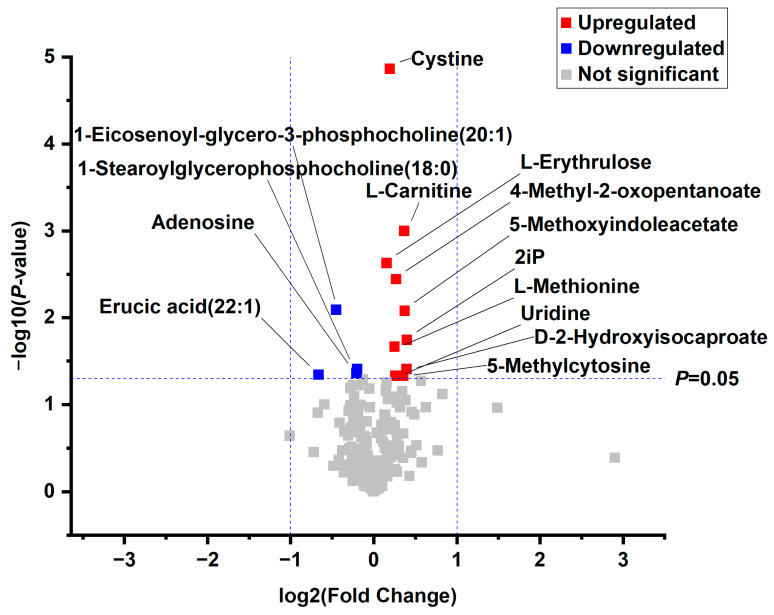
**Serum Metabolomics Changes Following PEM Treatment in Patients with Hypertriglyceridemia.** A volcano plot showing significantly altered serum metabolites in 7 patients after PEM treatment. Red and blue square represent significantly increased and decreased metabolites, respectively (*p* < 0.05), whereas gray square indicate metabolites without significant changes. The *x*-axis represents log2 fold change and the *y*-axis represents −log10 *p* value. The vertical dashed lines indicate the fold-change threshold (log2 fold change = ±1), and the horizontal dashed line indicates the significance threshold (*p* = 0.05).

**Figure 2:**
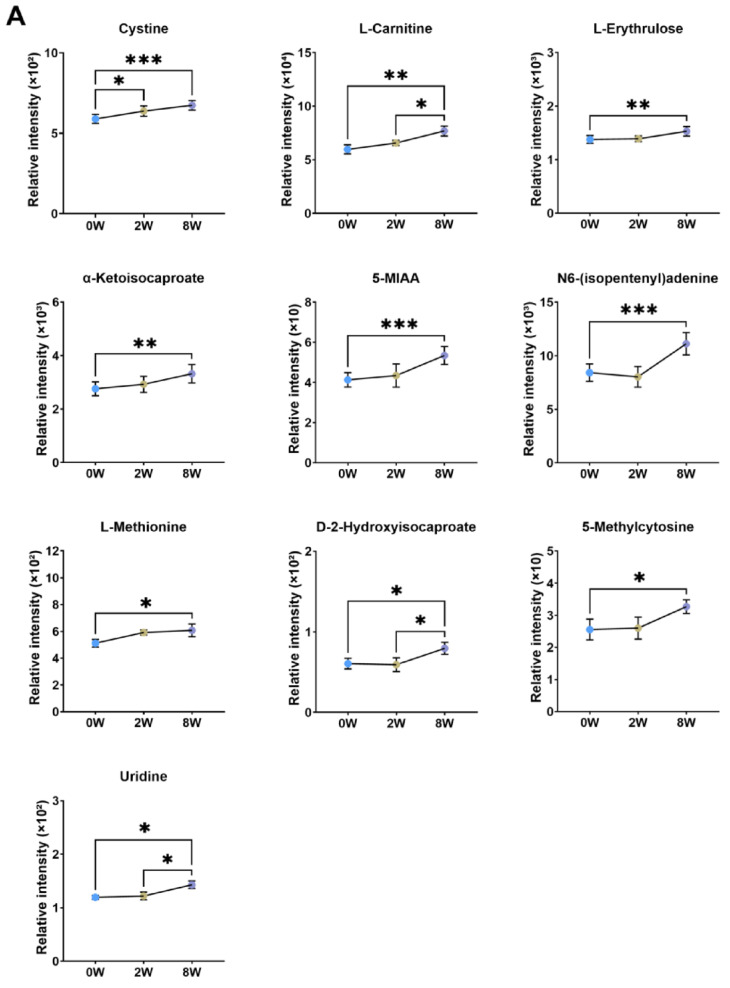

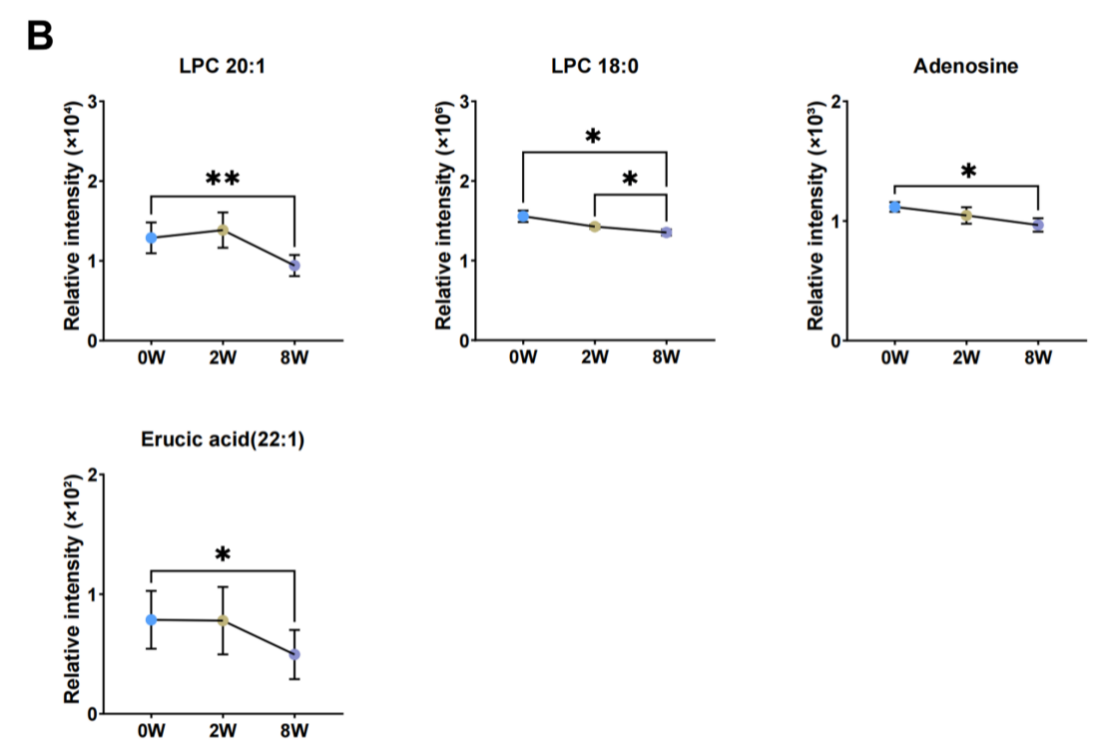
**Time Course of Significantly Altered Serum Metabolites Following PEM Treatment for 2 and 8 Weeks.** (**A**) Increased metabolites after 8-week (8 W) treatment. (**B**) Decreased metabolites after 8 W treatment. Data are presented as mean ± SEM. Statistical significance was evaluated by paired two-tailed Student’s *t*-test (* *p* < 0.05, ** *p* < 0.01, *** *p* < 0.001). 2 W, 2-week treatment; 5-MIAA, 5-methoxyindoleacetic acid; LPC, lysophosphatidylcholine.

To further characterize the temporal progression of metabolic alterations, serum metabolites at week 8 were compared with baseline. Several metabolites were significantly increased at week 8, including cystine, L-carnitine, L-erythrulose, L-methionine, and uridine, whereas others were significantly decreased, such as adenosine, erucic acid (22:1), and LPCs (Figure 1 and Figure S5 and Supplementary Tables S4 and S5). Time-course analysis further showed that L-carnitine, L-methionine, uridine, cystine, L-erythrulose, and 5-methoxyindoleacetate increased progressively from baseline to week 8, whereas adenosine, erucic acid (22:1), and LPC species showed sustained decreases over time (Figure 2 and Figures S2–S4). Together, these results indicate that PEM induces time-dependent changes in the human serum metabolome, characterized by increases in carnitine and amino acid-related metabolites and decreases in lipid-related metabolites.

### 2.2. Circulating Carnitine Levels Are Increased and Ketone Bodies Show Increasing Trends After PEM Treatment in Patients with Hypertriglyceridemia

**Figure 3:**
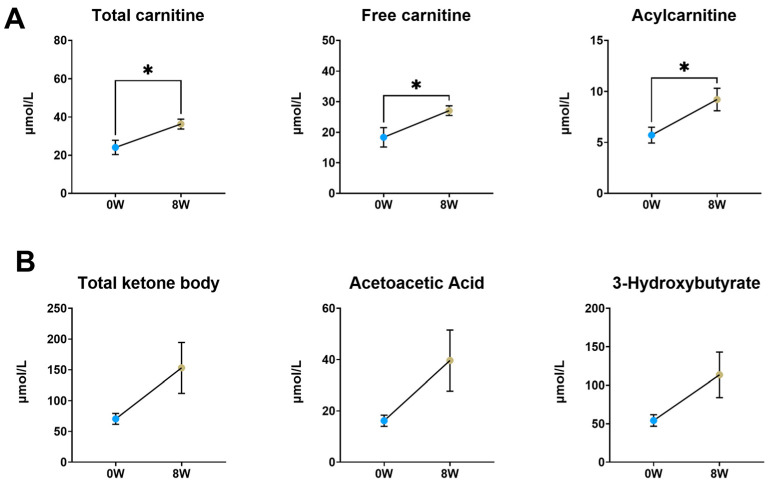
**Serum Carnitine Levels and Ketone Bodies After PEM Treatment.** Serum samples used for a metabolomic analysis were assayed at baseline (0 W) and after 8-week treatment (8 W). (**A**) Total carnitine, free carnitine, and acylcarnitine levels (*n* = 5). (**B**) Total ketone bodies, acetoacetic acid, and 3-hydroxybutyrate levels (*n* = 7). Due to limited sample volume available, carnitine measurements were performed in a subset of subjects with sufficient remaining serum samples. Data are presented as mean ± SEM. Statistical significance was evaluated by paired two-tailed Student’s *t*-test (* *p* < 0.05).

Untargeted serum metabolomic analysis revealed an increase in carnitine following PEM treatment. To confirm this finding, serum carnitine levels were further measured using a different enzymatic assay. Due to limited sample volume available, carnitine measurements were performed in a subset of subjects with sufficient remaining serum samples (*n* = 5). In these subjects, PEM treatment significantly increased total carnitine, free carnitine, and acylcarnitine levels at week 8 compared with baseline (Figure 3A and Figure S6A).

Because carnitine is required for mitochondrial fatty acid transport and β-oxidation, we examined whether changes in carnitine after PEM treatment were associated with changes in circulating ketone bodies, which are commonly used as indicators of hepatic fatty acid β-oxidation. Total ketone bodies, acetoacetic acid, and 3-hydroxybutyrate in serum showed upward trends after PEM treatment, although these changes did not reach statistical significance (Figure 3B and Figure S6B).

Together, these results indicate that PEM increases circulating carnitine levels in patients with hypertriglyceridemia, whereas ketone bodies show only modest, non-significant increasing trends.

### 2.3. A Clinically Relevant Dose of PEM Increases Circulating and Tissue Carnitine Levels in Mice

**Figure 4:**
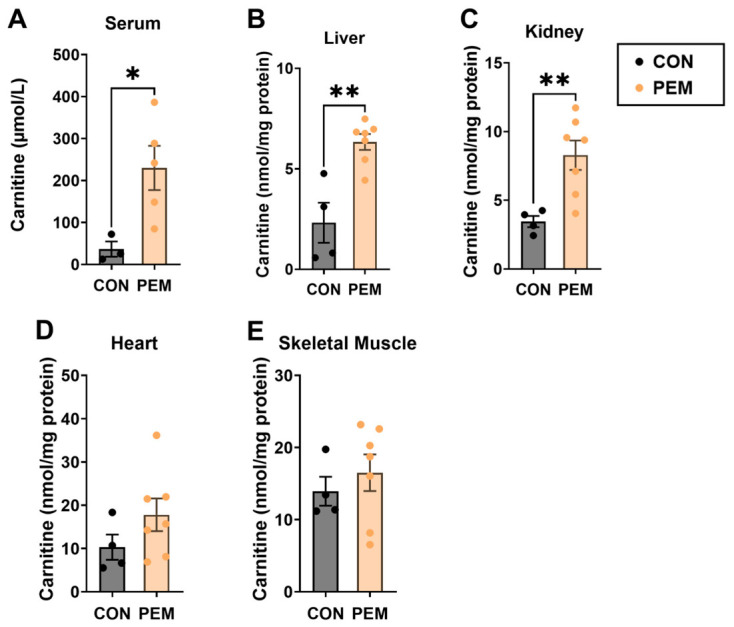
**Serum and Tissue Carnitine Levels in Mice Treated with a Clinically Relevant Dose of PEM** (**0.1 mg/kg/day**) **for 2 Weeks.** Carnitine levels were measured using archived samples from the previous study [9]. In the previous study, PEM was suspended in 0.5% (*w*/*v*) carboxymethylcellulose at final concentrations of 0.01 mg/mL for the 0.1 mg/kg/day treatment, and the suspension was administered by oral gavage once daily at approximately 10:00 am for 2 weeks with male 8-week-old mice on a C57BL/6J genetic background weighing 20–25 g. The suspension without PEM was administered to the control group (CON). (**A**–**E**) Carnitine levels of serum (**A**) (CON, *n* = 3; PEM, *n* = 5), liver (**B**), kidney (**C**), heart (**D**), and skeletal muscle (**E**) (CON, *n* = 4; PEM, *n* = 7). Data are shown as mean ± SEM. Statistical significance was evaluated by unpaired two-tailed Student’s *t*-test (* *p* < 0.05, ** *p* < 0.01). CON, vehicle-treated control mice; PEM, PEM-treated mice. The number of serum samples was small because of the limited sample volume available.

To examine whether similar alterations in carnitine metabolism are observed in an experimental model, we measured carnitine concentrations in serum and multiple tissues in mice treated with a clinically relevant dose of PEM. For this analysis, we used archived serum and tissue samples from our previously reported mouse study [9]. PEM treatment significantly increased serum carnitine levels compared with the control group (Figure 4A). In addition, carnitine levels (normalized to protein content) were significantly elevated in the liver (Figure 4B) and kidney (Figure 4C) of PEM-treated mice. Carnitine levels in the heart (Figure 4D) and skeletal muscle (Figure 4E) also tended to be higher after PEM treatment. Importantly, this trend remained unchanged after normalization to tissue weight (Supplementary Figure S7). Taken together, these results suggest that PEM treatment is associated with increased circulating and tissue carnitine levels in mice.

### 2.4. A Clinically Relevant Dose of PEM Does Not Alter Carnitine Transporter Expression in Mice

**Figure 5:**
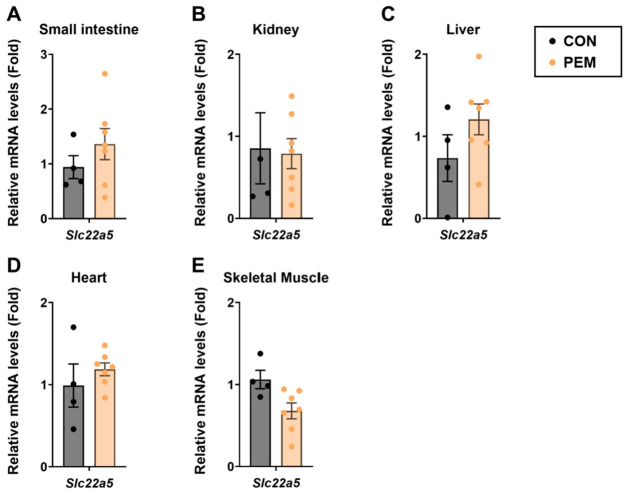
***Slc22a5* mRNA Expression in Mice Treated with a Clinically Relevant Dose of PEM** (**0.1 mg/kg/day**) **for 2 Weeks.** Available tissue samples from the same mouse experiment were subjected to quantitative PCR (qPCR) analysis. (**A**–**E**) The *Slc22a5* mRNA levels in small intestine (**A**), kidney (**B**), liver (**C**), heart (**D**), and skeletal muscle (**E**), respectively. The mRNA levels were normalized to that of 18S rRNA and expressed as values relative to those of control mice. Data are shown as mean ± SEM. CON, vehicle-treated control mice (*n* = 4); PEM, PEM-treated mice (*n* = 7).

To determine whether the PEM-induced increase in tissue carnitine levels was related to altered carnitine transport, we next examined the expression of the major carnitine transporter organic cation/carnitine transporter 2 (OCTN2). OCTN2, encoded by *Slc22a5*, is a main Na^+^-dependent carnitine transporter responsible for intestinal absorption, renal reabsorption, and tissue uptake of carnitine [10]. To examine whether the PEM-induced increase in tissue carnitine levels was associated with altered carnitine transport, *Slc22a5* mRNA levels were measured in the small intestine, kidney, liver, heart, and skeletal muscle with quantitative PCR (qPCR) analysis. *Slc22a5* mRNA expression was not significantly different between control and PEM-treated mice in any of the examined tissues (Figure 5). These results indicate that the increase in tissue carnitine levels observed after PEM treatment is unlikely due to upregulation of *Slc22a5*-mediated carnitine transport.

### 2.5. PEM Has Limited Effects on Carnitine Biosynthesis-Related Gene Expression in Mice

**Figure 6:**
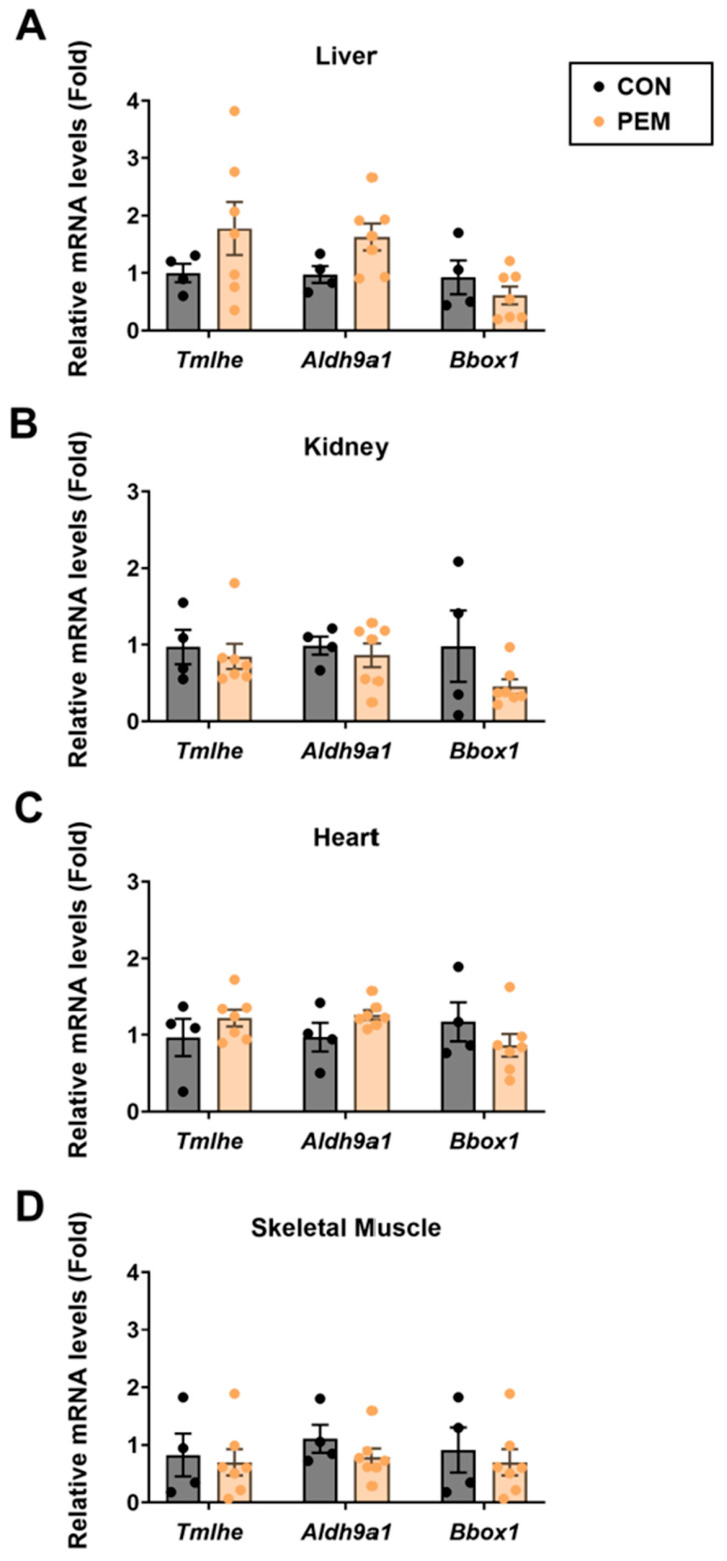
**The mRNA Levels of Carnitine Biosynthesis-Related Genes in Mice Treated with a Clinically Relevant Dose of PEM** (**0.1 mg/kg/day**) **for 2 Weeks.** Available tissue samples from the same mouse experiment were used and the mRNA levels of trimethyllysine hydroxylase *(Tmlhe*), aldehyde dehydrogenase 9 family member A1 (*Aldh9a1*), and γ-butyrobetaine dioxygenase (*Bbox1*) were determined in the liver (**A**), kidney (**B**), heart (**C**), and skeletal muscle (**D**), respectively. The mRNA levels were normalized to that of 18S rRNA and expressed as values relative to those of control mice. Data are shown as mean ± SEM. CON, vehicle-treated control mice (*n* = 4); PEM, PEM-treated mice (*n* = 7).

Carnitine can be synthesized endogenously from lysine and L-methionine through a multistep pathway, with *de novo* biosynthesis occurring primarily in the liver and kidney [11]. To examine whether PEM affects endogenous carnitine biosynthesis, the mRNA levels of trimethyllysine hydroxylase (*Tmlhe*), aldehyde dehydrogenase 9 family member A1 (*Aldh9a1*), and γ-butyrobetaine dioxygenase (*Bbox1*) were measured in the liver, kidney, heart, and skeletal muscle. PEM treatment did not result in consistent changes in the expression of these genes in these tissues of mice (Figure 6).

### 2.6. PEM-Induced Carnitine Increases Are Associated with Carnitine Acyltransferases in Mice

**Figure 7:**
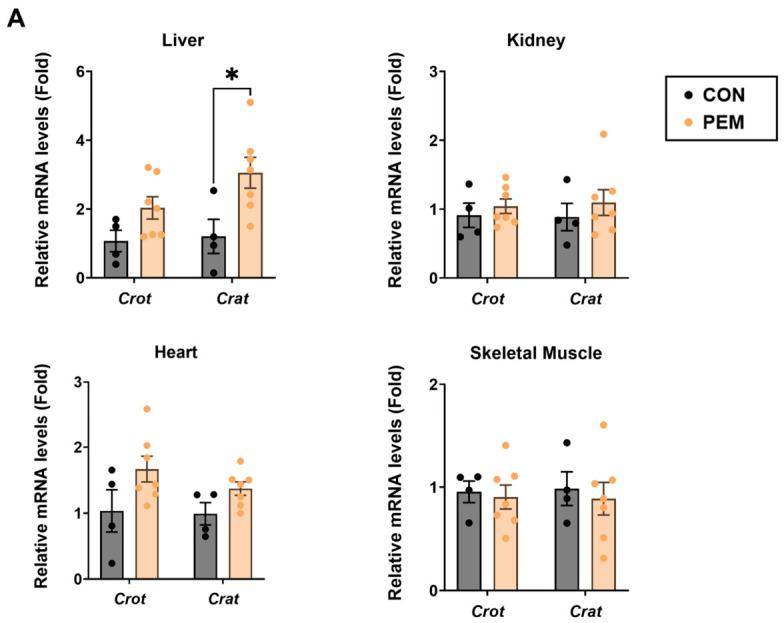

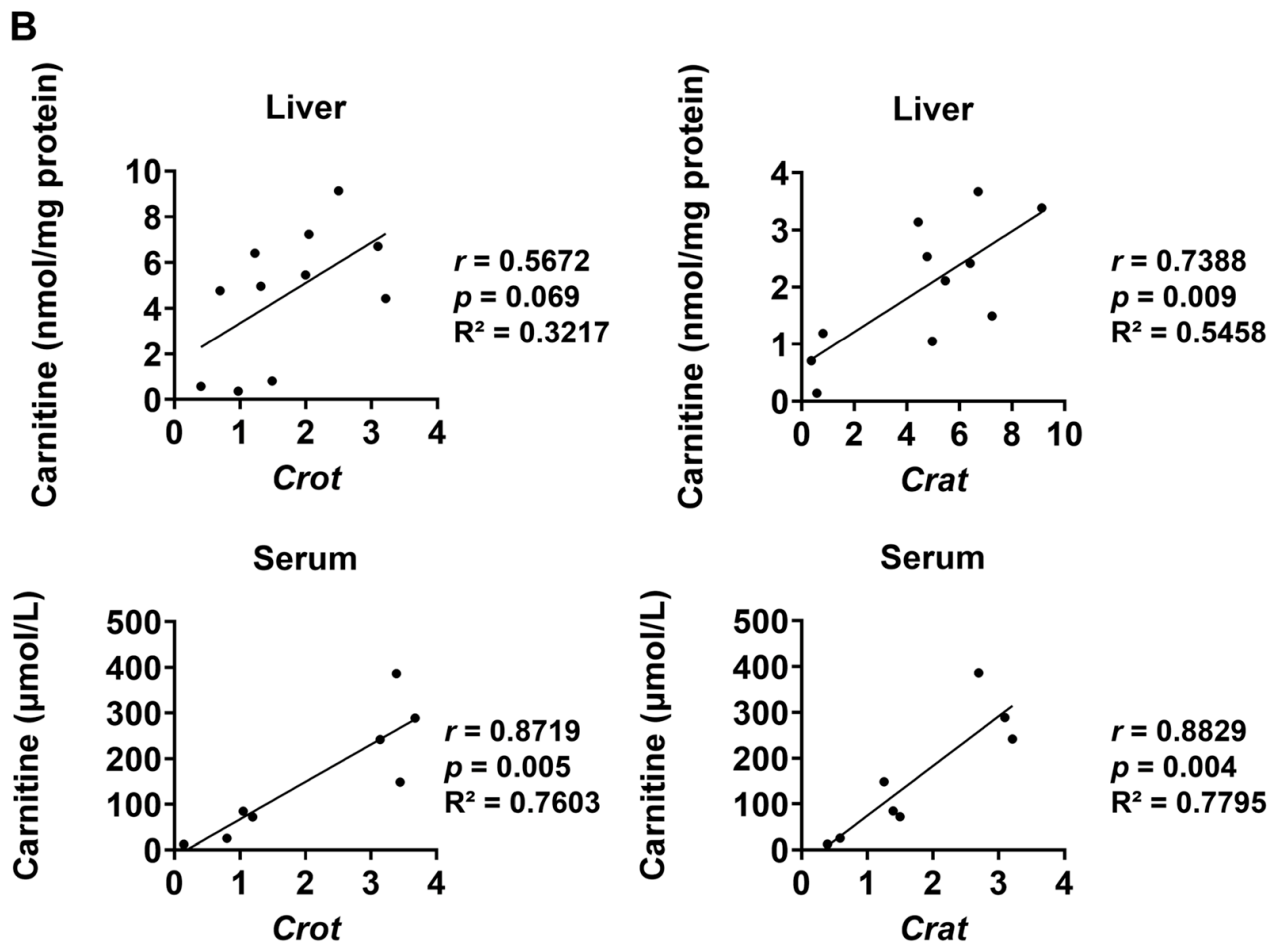
**The mRNA Levels of Carnitine Acyltransferase Genes in Mice Treated with a Clinically Relevant Dose of PEM (0.1 mg/kg/day) for 2 Weeks**. (**A**) Available tissue samples from the same mouse experiment were used and the mRNA levels of carnitine O-octanoyltransferase (*Crot*) and carnitine O-acetyltransferase (*Crat*) were quantified in the liver, kidney, heart, and skeletal muscle, respectively. The mRNA levels were normalized to that of 18S rRNA and expressed as values relative to those of control mice. Data are shown as mean ± SEM. Statistical significance was evaluated by unpaired two-tailed Student’s *t*-test (* *p* < 0.05). CON, vehicle-treated control mice (*n* = 4); PEM, PEM-treated mice (*n* = 7). (**B**) Correlation between hepatic *Crot* or *Crat* mRNA levels and carnitine concentrations in liver and serum. Correlation coefficients were calculated using Pearson’s test. The samples shown in Figure 4 were used (*n* = 4 and 7 in CON and PEM, respectively, for the correlation analysis of liver samples: *n* = 3 and 5 in CON and PEM, respectively, for the correlation analysis of serum samples).

Carnitine acyltransferases, including carnitine O-octanoyltransferase (CROT, encoded by *Crot*) and carnitine O-acetyltransferase (CRAT, encoded by *Crat*), are involved in acyl group transfer between free carnitine and acylcarnitines in peroxisomes and mitochondria, respectively [12,13]. To examine the effects of PEM on genes involved in carnitine utilization, the mRNA levels of *Crot* and *Crat* were quantified in the liver, kidney, heart, and skeletal muscle. PEM treatment significantly increased the mRNA levels of *Crot* and *Crat* in the liver, whereas no significant changes were observed in the kidney, heart, or skeletal muscle (Figure 7A). Furthermore, hepatic *Crot* and *Crat* mRNA levels showed positive associations with carnitine levels in liver tissue and serum, although the correlation between *Crot* mRNA and liver carnitine did not reach statistical significance (Figure 7B). Therefore, hepatic upregulation of *Crot*/*Crat* mRNA may be associated with PEM-induced increases in carnitine levels in mice.

### 2.7. Effects of PEM on the Mitochondrial Carnitine Shuttle in Mice

**Figure 8:**
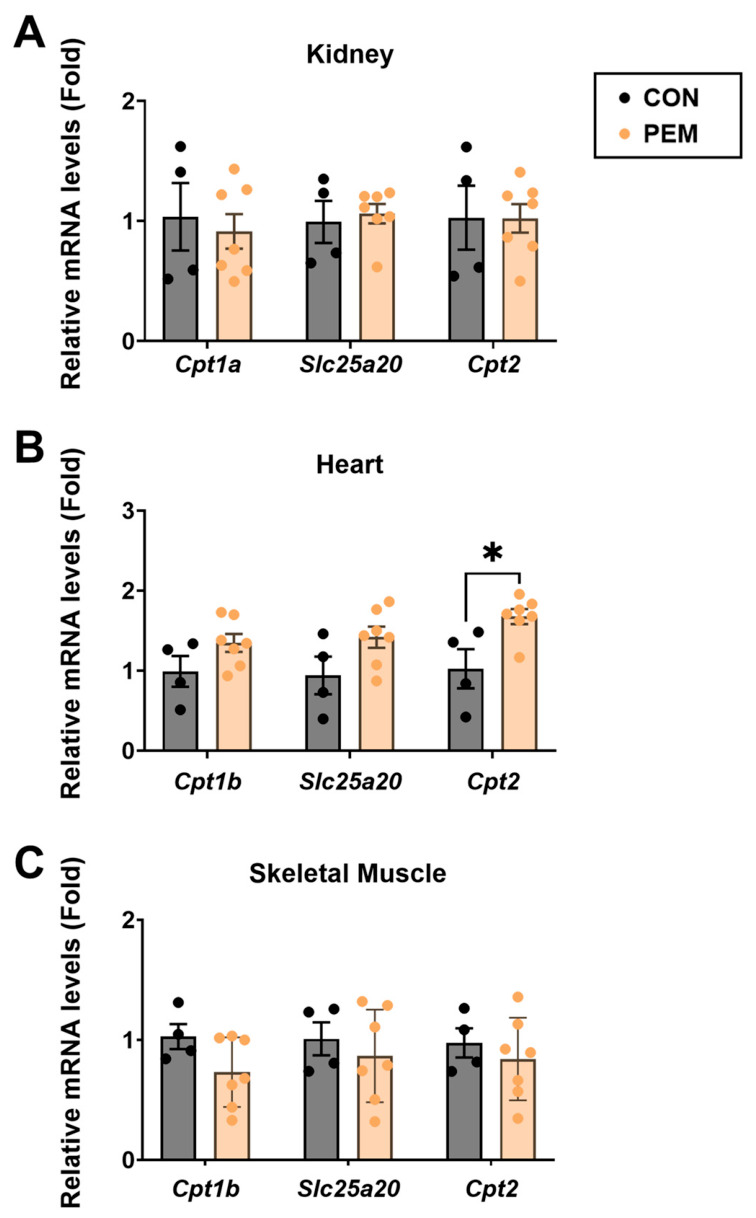
**The mRNA Levels of Genes Involved in the Mitochondrial Carnitine Shuttle in Extrahepatic Tissues of Mice Treated with a Clinically Relevant Dose of PEM** (**0.1 mg/kg/day**) **for 2 Weeks.** The same samples in Figure 5 were used and the mRNA levels of carnitine palmitoyltransferase 1a (*Cpt1a*) in the kidney (**A**), carnitine palmitoyltransferase 1b (*Cpt1b*) in the heart (**B**) and skeletal muscle (**C**), as well as carnitine/acylcarnitine translocase (*Slc25a20*) and carnitine palmitoyltransferase 2 (*Cpt2*), were assessed. The mRNA levels were normalized to that of 18S rRNA and expressed as values relative to those of control mice. Data are shown as mean ± SEM. Statistical significance was evaluated by unpaired two-tailed Student’s *t*-test (* *p* < 0.05). CON, vehicle-treated control mice (*n* = 4); PEM, PEM-treated mice (*n* = 7).

We previously demonstrated that a clinically relevant dose of PEM enhanced the mRNA levels of mitochondrial carnitine/acylcarnitine translocase (encoded by *Slc25a20*), but not carnitine palmitoyltransferase 1 and 2 [encoded by carnitine palmitoyltransferase 1a (*Cpt1a*) or *Cpt2*] in mouse livers [9]. In addition to the liver, we examined the expression of genes associated with carnitine shuttle through mitochondrial membrane in extrahepatic tissues. Since carnitine palmitoyltransferase 1b (*Cpt1b*) is the predominant isoform in skeletal muscle, we assessed its mRNA levels in the heart and skeletal muscle, while *Cpt1a* (liver-type isoform) was measured in the kidney, as it is the major isoform expressed in that organ. PEM treatment had limited and tissue-specific effects on the expression of *Cpt1a*, *Cpt1b*, *Slc25a20*, and *Cpt2* genes in the examined tissues (Figure 8).

## 3. Discussion

This study investigated how PEM altered serum metabolites in patients with hypertriglyceridemia. Untargeted metabolomics uncovered that PEM treatment was associated with coordinated changes in multiple metabolites, including increased levels of cystine, L-methionine, and uridine, and decreased levels of lipid-related metabolites such as LPC, indicating changes in multiple metabolic pathways by PEM. Among them, carnitine metabolism was markedly altered by PEM treatment in patients with hypertriglyceridemia, showing a clear increase over time. To our knowledge, this is the first study to characterize systemic metabolic effects of PEM using an untargeted metabolomic approach.

The most intriguing finding in this study was that circulating carnitine levels showed a clear and consistent increase following the treatment of PEM in clinical situations. In contrast, ketone body levels showed only modest, non-significant increasing trends. Although previous studies have shown that treatment with potent PPARα agonists or fasting conditions typically leads to marked changes in metabolites related to fatty acid β-oxidation [14], the results of the present study indicate that increased carnitine is a main metabolic pathway altered by PEM administration, preceding enhanced fatty acid β-oxidation. In addition, PEM impacts other metabolites in serum, such as cystine, L-erythrulose, 5-methylcytosine, and uridine, which are related to β-oxidation, oxidative stress, and amino acid metabolism. Although the principal effect of PEM may be mediated by carnitine metabolism, PEM clearly modifies several metabolic pathways.

To explore the mechanism by which PEM enhances serum carnitine levels in humans, we examined whole-body carnitine metabolism in mice treated with a clinically relevant dose of PEM. PEM treatment not only increased serum carnitine levels in mice but also elevated carnitine levels in multiple tissues, including the liver, kidney, heart, and skeletal muscle, indicating that the increase in carnitine was not restricted to a single organ. However, under the same conditions, no global upregulation of carnitine *de novo* biosynthesis in each tissue or uptake from the intestine was observed, corroborating the increase in circulating and tissue carnitine is likely associated with upregulation of CROT and CRAT expression in the liver [12,13].

Hepatic expressions of CROT and CRAT showedpositive correlations with carnitine levels in both liver and serum in mice. Consistent with this interpretation, a previous study has shown that the disruption of *Crot* markedly reduces hepatic carnitine levels in mice [12], supporting the role of CROT in tissue carnitine regulation. In contrast, CRAT regulates the balance between acetyl-CoA and acetylcarnitine [13], suggesting that it may influence carnitine utilization rather than the total levels. Additionally, previous studies have demonstrated in cellular experiments that fenofibrate, a PPARα agonist, and L-carnitine synergistically enhanced *Crat* mRNA expression [5]. Our findings are consistent with these observations, suggesting that PEM up-regulates *Crat/Crot* expression presumably through PPARα activation.

A recent post hoc analysis of a randomized, placebo-controlled trial involving 118 patients with metabolic dysfunction-associated steatotic liver disease (MASLD) documented that PEM treatment significantly increased serum total and free carnitine levels compared with placebo, with a trend toward increased acylcarnitines [15]. In contrast, the present study systematically assessed time-course changes in serum metabolites using an untargeted metabolomic approach and identified carnitine as a significantly increased metabolite after PEM treatment, complemented by mechanistic investigations in a mouse model. Basically, metabolomics from any biofluid tends to be noisy, especially in human samples. The participants of the present study had different backgrounds and were not provided with standardized lifestyle interventions during the treatment period. Even in such highly variable circumstances, it is meaningful that carnitine was identified as a remarkable metabolite altered by PEM treatment. Consistency of results from the two studies, the previous randomized controlled trial [15] and the present untargeted metabolomic analysis clearly corroborates the significance of carnitine metabolism as an important axis of PEM’s action.

Previous studies have demonstrated that carnitine deficiency is caused by congenital disorders, such as OCTN2 mutation, liver cirrhosis, and routine hemodialysis, and the restoration of carnitine homeostasis can alleviate hepatic lipid accumulation and improve some typical symptoms, such as muscle cramp [10,16]. Moreover, multiple clinical and animal studies have shown that PEM lowers serum triglyceride levels and improves hepatic lipid accumulation and liver function markers in MASLD and metabolic dysfunction-associated steatohepatitis (MASH) [17,18]. PEM-induced carnitine increases may be associated with its metabolic benefits in MASLD/MASH and related disorders [17,18].

In the present study, we further observed that PEM treatment significantly increased circulating free carnitine and acylcarnitines in association with hepatic upregulation of *Crot* and *Crat* mRNA and enhancing the carnitine–acylcarnitine buffering system, potentially contributing to intracellular acyl-group redistribution and lipid handling [10,19]. Additionally, PEM seemed to enhance carnitine turnover in the liver, release into circulation, and supply carnitine from liver to the other tissues, such as kidney, heart, and skeletal muscle. The detailed changes in PEM-induced systemic carnitine metabolism and its precise mechanism should be assessed in further studies.

This study has several limitations. First, the human cohort of this study was small, with seven subjects included in the metabolomic analysis and five subjects available for enzymatic carnitine measurements because of limited remaining serum volume available. Additionally, all participants in the human cohort were female. However, PEM also increased circulating and tissue carnitine levels in male mice, suggesting that this finding may not be associated with sexual and species differences. Larger placebo-controlled studies are needed to validate PEM-induced changes in circulating carnitine and related metabolites. Second, because CROT/CRAT were evaluated only at the mRNA level, future studies should assess enzymatic activities of hepatic CROT/CRAT. Third, there was the relatively small sample size in the animal experiment. In accordance with the principles of Replacement, Reduction, and Refinement (3Rs), animal numbers were minimized while maintaining statistical rigor and achieving the scientific objectives. Although the number of animals was relatively small, statistically significant differences were consistently observed across multiple independent endpoints using appropriate statistical analyses. These findings supported our conclusions, but validation in larger cohorts would be valuable in future studies. And lastly, future studies using liver-specific *Ppara*- and/or *Crot/Crat*-disrupted mice would be useful to confirm whether PEM-induced changes in carnitine metabolism are mediated through the hepatic PPARα-CROT/CRAT axis.

## 4. Materials and Methods

### 4.1. Human Serum Sample Collection

Serum samples were obtained from seven patients with hypertriglyceridemia (Patients 1–7) at baseline (0 weeks) and after 2 and 8 weeks of PEM treatment (0.2 mg/day) at Shinshu University Hospital (Matsumoto, Japan) and the affiliated hospitals. Baseline characteristics of the study participants are summarized in Supplementary Table S1. Serum samples were drawn after an overnight fast, allowed to clot at room temperature, and subsequently centrifuged at 3000× *g* for 15 min. Participants were not provided with a standardized diet during the treatment period. The resulting serum was carefully collected and stored at −80 °C until further analysis. Written informed consent was obtained from all participants prior to sample collection. The study was conducted in accordance with the Declaration of Helsinki and was approved by the Ethics Committee of Shinshu University School of Medicine [Approval ID #4196 (23 October 2018) and #6682 (28 November 2025)].

### 4.2. Untargeted Serum Metabolomic Analysis

For metabolomic analysis, 50 μL of each serum sample was mixed with 200 μL of methanol containing exogenous internal standards for signal correction and vortexed vigorously to precipitate proteins. After the addition of 200 μL of ultrapure water, the mixture was centrifuged at 2300× *g* for 5 min at 4 °C. The supernatant was filtered using a 5-kDa cutoff centrifugal filter (Millipore, Billerica, MA, USA) to remove residual macromolecules. The filtrate was dried under vacuum and reconstituted in 50 μL of ultrapure water. Untargeted metabolomic profiling was performed using a combination of capillary electrophoresis–time-of-flight mass spectrometry (CE-TOFMS) and liquid chromatography–time-of-flight mass spectrometry (LC-TOFMS). CE-TOFMS was used to analyze ionic and highly polar metabolites, whereas LC-TOFMS targeted neutral and hydrophobic compounds. All measurements were carried out by Human Metabolome Technologies, Inc. (HMT, Tsuruoka, Japan).

CE-TOFMS analysis was performed using an Agilent CE system equipped with an Agilent 6210 TOF mass spectrometer (Agilent Technologies, Santa Clara, CA, USA), and LC-TOFMS analysis was conducted using an Agilent 1200 series HPLC system coupled to the same TOFMS. Both analyses were performed in positive and negative ionization modes over an *m*/*z* range of 50–1000. CE-TOFMS and LC-TOFMS analyses were performed according to the standard analytical protocol of Human Metabolome Technologies, Inc. Detailed CE-TOFMS and LC-TOFMS analytical conditions were based on the previously described protocol [20].

Both data acquisition and raw data processing were performed using Agilent MassHunter Workstation B.04.01 software. which automatically extracts and quantifies peaks based on *m*/*z*, migration time (in CE-TOFMS), or retention time (in LC-TOFMS). Pooled quality control (QC) samples were analyzed throughout each analytical batch at regular intervals, with one QC sample analyzed per five study samples. Metabolites with a coefficient of variation greater than 50% across pooled QC samples were excluded from subsequent analyses. In addition, five exogenous internal standards were used for retention time, mass accuracy, and signal intensity correction. Batch effects between analytical runs were corrected using the median signal intensity of pooled QC samples. Metabolite identification was performed in accordance with the Metabolomics Standards Initiative (MSI) framework. For metabolite annotation, an in-house library consisting of approximately 1300 authentic chemical standards analyzed under the same CE-MS and LC-MS conditions as the study samples was used. Metabolites were identified by matching both accurate mass (*m*/*z*) and normalized retention/migration time with those of authentic standards. In cases where multiple candidate metabolites shared similar *m*/*z* and retention characteristics, additional MS/MS analyses were performed to confirm metabolite identity. Metabolites identified by matching both accurate mass and retention/migration time to authentic standards were assigned to MSI level 1. Metabolite intensities were normalized to internal standards to correct for technical variability.

### 4.3. Mice and Treatment

Mouse serum and tissue samples used in the previous study [9] were adopted for subsequent analyses. Briefly, male 8-week-old C57BL/6J mice weighing 20–25 g were purchased from CLEA Japan, Inc. (Tokyo, Japan) and were housed under controlled conditions (25 °C, 12 h light/dark cycle, 4–6 mice per cage) with free access to tap water and standard laboratory chow. After acclimatization, mice were randomly assigned to two groups: a vehicle-treated control group (CON) and a PEM-treated group (PEM). PEM (Kowa Company, Ltd., Tokyo, Japan) was suspended in 0.5% (*w*/*v*) carboxymethylcellulose (Wako Pure Chemical Industries, Osaka, Japan) and administered at a dose of 0.1 mg/kg/day, which was selected as a clinically relevant dose based on a previous study [9]. The control group received an equivalent volume of 0.5% carboxymethylcellulose alone. Drug or vehicle suspensions were administered once daily by oral gavage at approximately 10:00 a.m. for 2 weeks. After 2 weeks of treatment, mice were fasted for 4 h before euthanasia under anesthesia. Serum was collected immediately after euthanasia, and serum was prepared by centrifugation at 3000× *g* for 20 min followed by 10,000× *g* for 5 min. The small intestine, liver, kidney, heart, and skeletal muscle were rapidly excised, snap-frozen in liquid nitrogen, and stored at −80 °C until analysis. Absolute tissue weights used for normalization are shown in Supplementary Table S6.

All animal procedures were approved by the Animal Ethics Committee of Shinshu University School of Medicine [Approval ID #290026 (1 August 2017) and #300037 (12 July 2018)] and were conducted in accordance with the Guidelines for Proper Conduct of Animal Experiments of the Science Council of Japan and the guidelines of the National Academy of Sciences.

### 4.4. Measurement of Carnitine Levels in Mouse Tissues

Mouse tissues, including the liver, kidney, heart, and skeletal muscle, were rinsed in ice-cold phosphate-buffered saline (PBS), blotted dry, and weighed. Each sample (up to 50 mg) was then homogenized in 250 μL of PBS using a probe-type ultrasonic homogenizer. The homogenates were centrifuged at 12,000× *g* for 10 min at 4 °C, and the resulting supernatants were collected for further analysis. Carnitine concentrations were determined using a commercial enzymatic assay kit (L-Carnitine Assay Kit, ELCR-100, BioAssay Systems, Hayward, CA, USA) based on colorimetric detection at 570 nm.

All reactions were carried out at 37 °C, and absorbance was measured using a microplate reader (SpectraMax^®^ iD3, Molecular Devices, San Jose, CA, USA). Protein concentration was determined using a BCA assay (Pierce, Rockford, IL, USA). Carnitine levels were normalized to protein content and expressed as nmol/mg protein (tissue weight-normalized values are shown in Supplementary Figure S7, and the corresponding tissue weights are shown in Supplementary Table S6). Appropriate negative controls and standard curves were included in each run to ensure assay reliability.

### 4.5. Analysis of mRNA Expression

Approximately 25 mg of small intestine, kidney, or liver tissue was homogenized, and total RNA was extracted using the NucleoSpin^®^ RNA Plus kit (MACHEREY-NAGEL, Düren, Germany) according to the manufacturer’s instructions. The same amount of heart and skeletal muscle tissue was homogenized in 0.5 mL of TRI Reagent (MOR Research Center, OH, USA) by sonication until no visible tissue fragments remained. After phase separation, the aqueous phase was mixed with an equal volume of 75% ethanol and loaded onto a NucleoSpin^®^ RNA Plus column for RNA purification. RNA purity and concentration were measured using a NanoDrop 2000 (Thermo Fisher Scientific, Waltham, MA, USA). Complementary DNA (cDNA) was synthesized using ReverTra Ace^®^ qPCR RT Master Mix (Toyobo, Osaka, Japan). qPCR was performed using THUNDERBIRD^®^ SYBR qPCR Mix (Toyobo) on a QuantStudio™ 3 Real-Time PCR System (Thermo Fisher Scientific, Waltham, MA, USA). Primer sequences were designed based on the NCBI BLAST database (https://blast.ncbi.nlm.nih.gov/Blast.cgi; accessed on 4 July 2026) and are listed in Supplementary Table S7. Relative mRNA expression levels were calculated by the comparative Ct method, normalized to 18S rRNA, and expressed as fold changes relative to those of the control mice.

### 4.6. Statistical Analysis

For metabolomic analyses, metabolite intensities were log-transformed prior to statistical analysis. Paired two-tailed Student’s *t*-test was used to assess metabolite changes between time points. For other experimental data, paired or unpaired two-tailed Student’s *t*-tests were used, as appropriate. Correlation analyses were performed using Pearson correlation analysis. All data are presented as the mean ± standard error of the mean (SEM). A *p* value of less than 0.05 was considered statistically significant.

## 5. Conclusions

Serum metabolomics revealed that PEM enhanced carnitine metabolism in humans. In mice treated with a clinically relevant dose of PEM, increased circulating and tissue carnitine levels were associated with upregulation of *Crat* and *Crot* mRNA in the liver, but not the upregulation of *de novo* carnitine biosynthesis or intestinal uptake. These findings indicate that the carnitine-related pathway is a possible central metabolic axis for PEM action.

## Data Availability

The metabolomics data supporting the findings of this study are provided as an accompanying Excel file submitted together with the revised manuscript. Further inquiries can be directed to the corresponding authors.

## Supplementary Materials

The following supporting information can be downloaded at: https://www.mdpi.com/article/10.3390/ijms27146252/s1.

## Abbreviations

cDNA, complementary DNA; CE-TOFMS, capillary electrophoresis–time-of-flight mass spectrometry; CPT1a, carnitine palmitoyltransferase 1A; CPT1b, carnitine palmitoyltransferase 1B; CPT2, carnitine palmitoyltransferase 2; CRAT, carnitine O-acetyltransferase; CROT, carnitine O-octanoyltransferase; LC-TOFMS, liquid chromatography–time-of-flight mass spectrometry; LPC, lysophosphatidylcholine; MASH, metabolic dysfunction-associated steatohepatitis; MASLD, metabolic dysfunction-associated steatotic liver disease; MSI, Metabolomics Standards Initiative; OCTN2, organic cation/carnitine transporter 2; PBS, phosphate-buffered saline; PEM, pemafibrate; PPARα, peroxisome proliferator-activated receptor α; QC, quality control; qPCR, quantitative polymerase chain reaction; SEM, standard error of the mean; SPPARMα, selective PPARα modulator.
