# Supplementary material for: Serum Metabolomics Reveals Carnitine Metabolism as a Possible Central Metabolic Axis of Pemafibrate Action
Source: Int J Mol Sci. 2026 Jul 14;27(14):6252. doi: 10.3390/ijms27146252 (PMC13411396; doi:10.3390/ijms27146252)
Supplement: Supplementary file 1 [file ijms-27-06252-s001.zip › ijms-4346185-supplementary.pdf]

Supporting Information

Index

Supplementary Figure S1
Time-course changes in serum lipid parameters following PEM treatment.

Supplementary Figure S2
Serum metabolite changes between baseline (0W) and 2 weeks (2W) following PEM treatment.

Supplementary Figure S3
Time-course changes in serum metabolites markedly altered by 2W PEM treatment.

Supplementary Figure S4
Individual patient trajectories of serum metabolites markedly altered by 2W PEM treatment.

Supplementary Figure S5
Individual patient trajectories of serum metabolites markedly altered by 8W PEM treatment.

Supplementary Figure S6
Individual changes in serum carnitine and ketone body concentrations from baseline (0W) to 8 weeks (8W) following PEM treatment.

Supplementary Figure S7
Carnitine concentrations (nmol/mg tissue) in mouse tissues following PEM treatment.

Supplementary Table S1
Baseline characteristics of the study participants.

Supplementary Table S2
Up-regulated metabolites by 2W PEM treatment.

Supplementary Table S3

Down-regulated metabolites by 2W PEM treatment.

Supplementary Table S4
Up-regulated metabolites by 8W PEM treatment.

Supplementary Table S5
Down-regulated metabolites by 8W PEM treatment.

Supplementary Table S6
Absolute tissue weights used for normalization of tissue carnitine concentrations.

Supplementary Table S7
Primer pairs used for qPCR analysis.

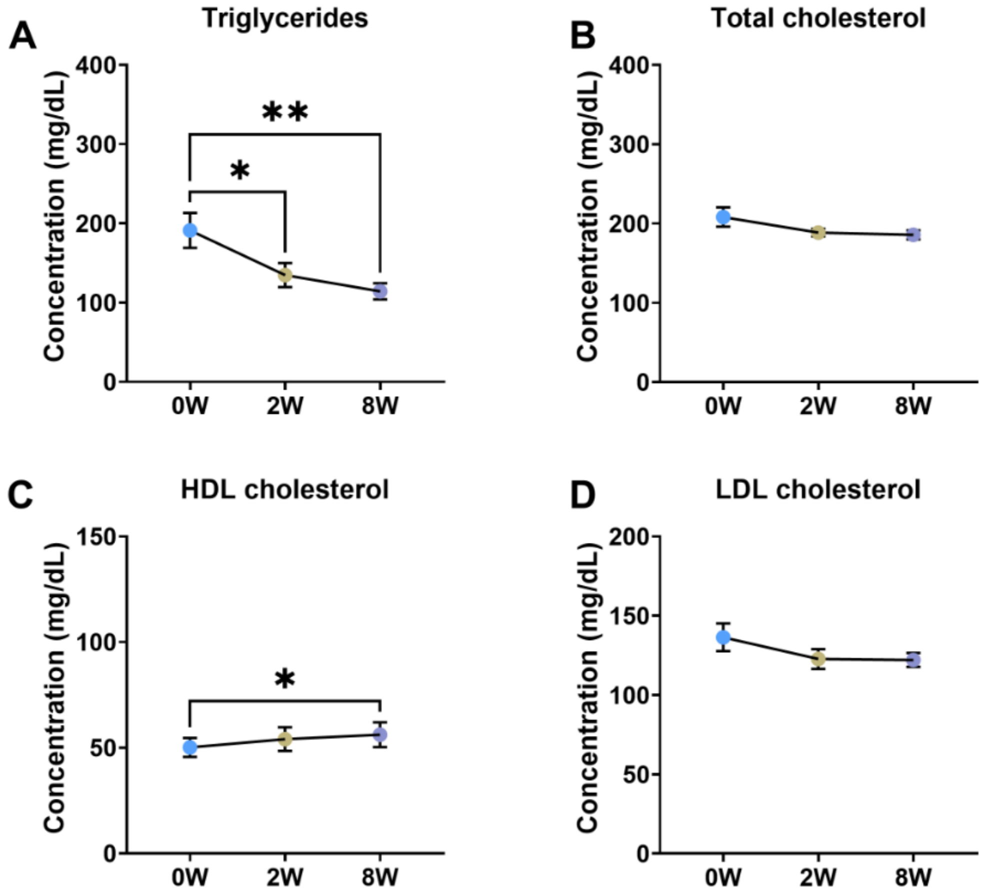

Figure S1. Time-course changes in serum lipid parameters following PEM treatment.

(A) Triglycerides, **(B)** Total cholesterol, **(C)** High-density-lipoprotein (HDL) cholesterol, and **(D)** Low-density-lipoprotein (LDL) cholesterol levels were measured at 0, 2, and 8 weeks after PEM treatment. Data are presented as mean ± SEM. Statistical significance was evaluated by paired two-tailed Student's *t*-test (*P<0.05, **P<0.01). W, weeks after commencing PEM treatment.

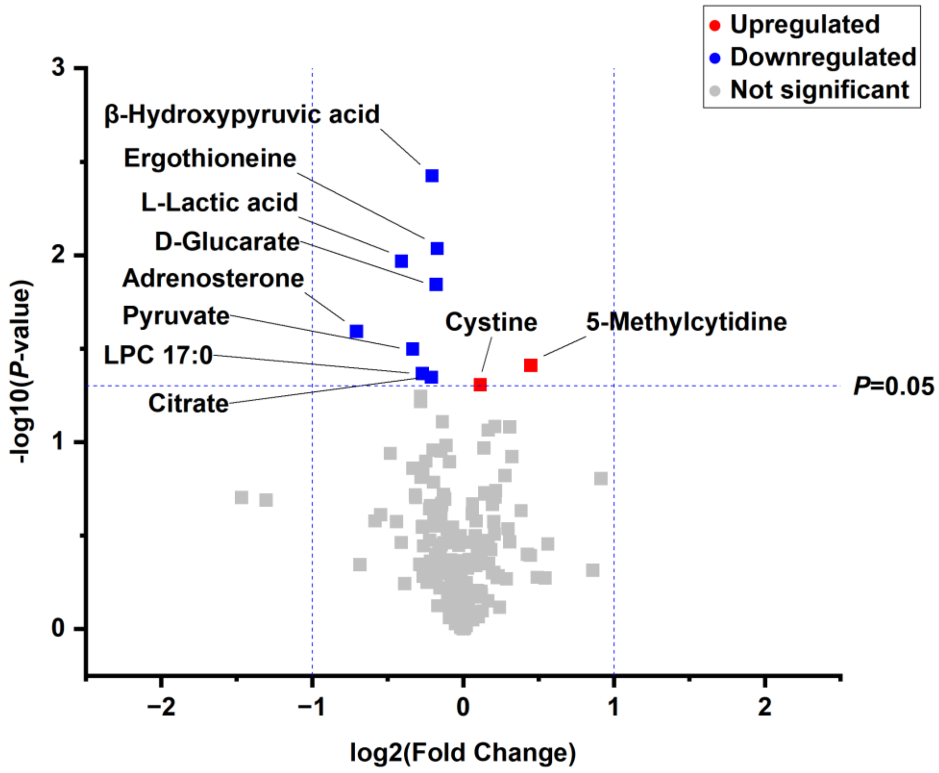

Figure S2. Serum metabolite changes between baseline (0W) and 2 weeks (2W) following PEM treatment.

A volcano plot showing significantly altered serum metabolites between baseline (0W) and 2 weeks (2W). Red and blue dots represent significantly increased and decreased metabolites, respectively (P<0.05), whereas gray dots indicate metabolites without significant changes. The x-axis represents $\log_2$ fold change, and the y-axis represents $-\log_{10} P$ value. The horizontal dashed line indicates the significance threshold (P=0.05).

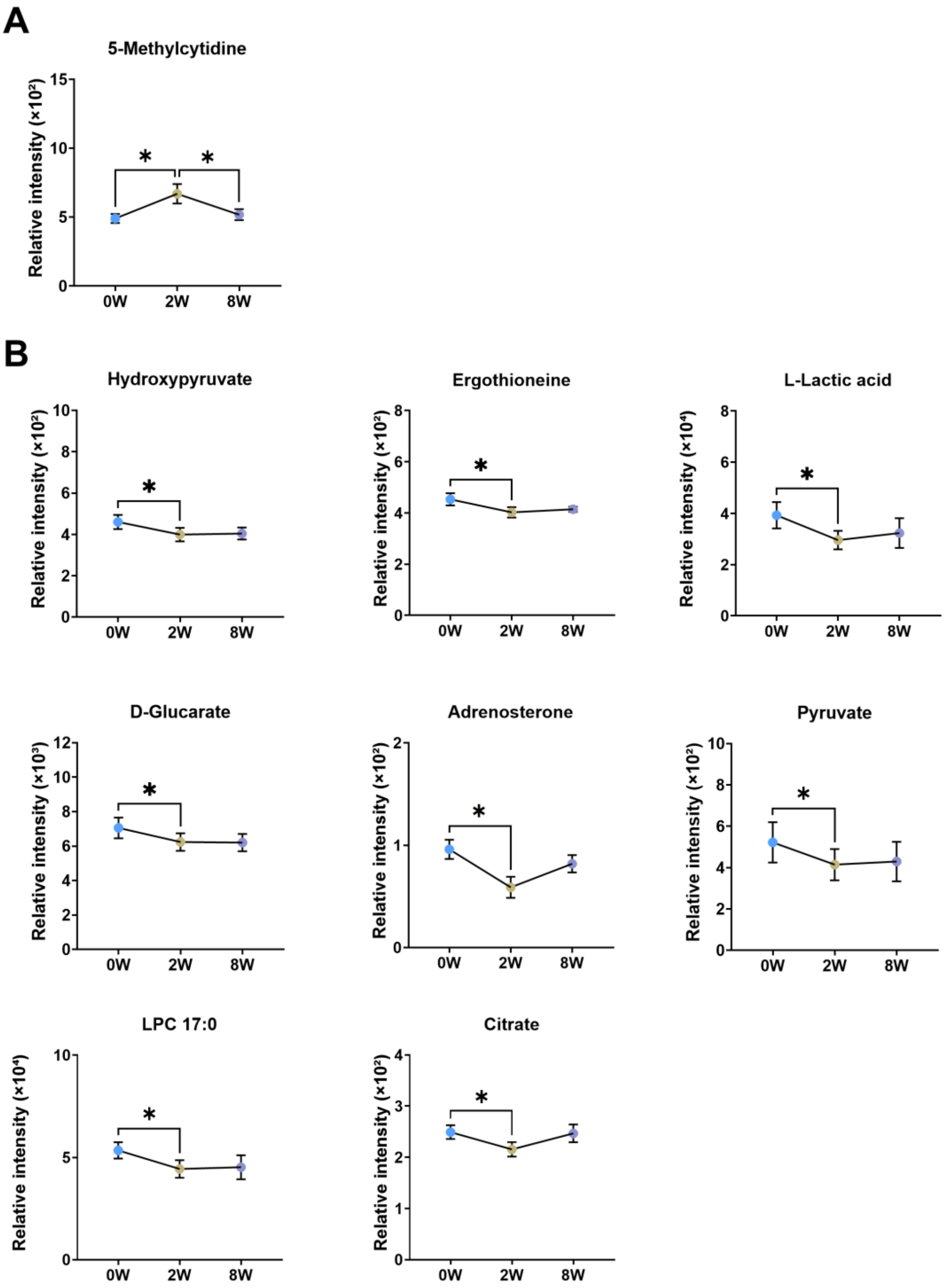

Figure S3. Time-course changes in serum metabolites markedly altered by 2W PEM treatment

(**A**) Time-course profiles of a metabolite significantly increased at 2 weeks (2W). (**B**) Time-course profiles of metabolites significantly decreased at 2W. Metabolite levels were measured at 0, 2, and 8 weeks and are presented as mean ± SEM (n = 7). Statistical significance at 2 weeks compared to baseline (0W) was evaluated by paired two-tailed Student's *t*-test (*P<0.05).

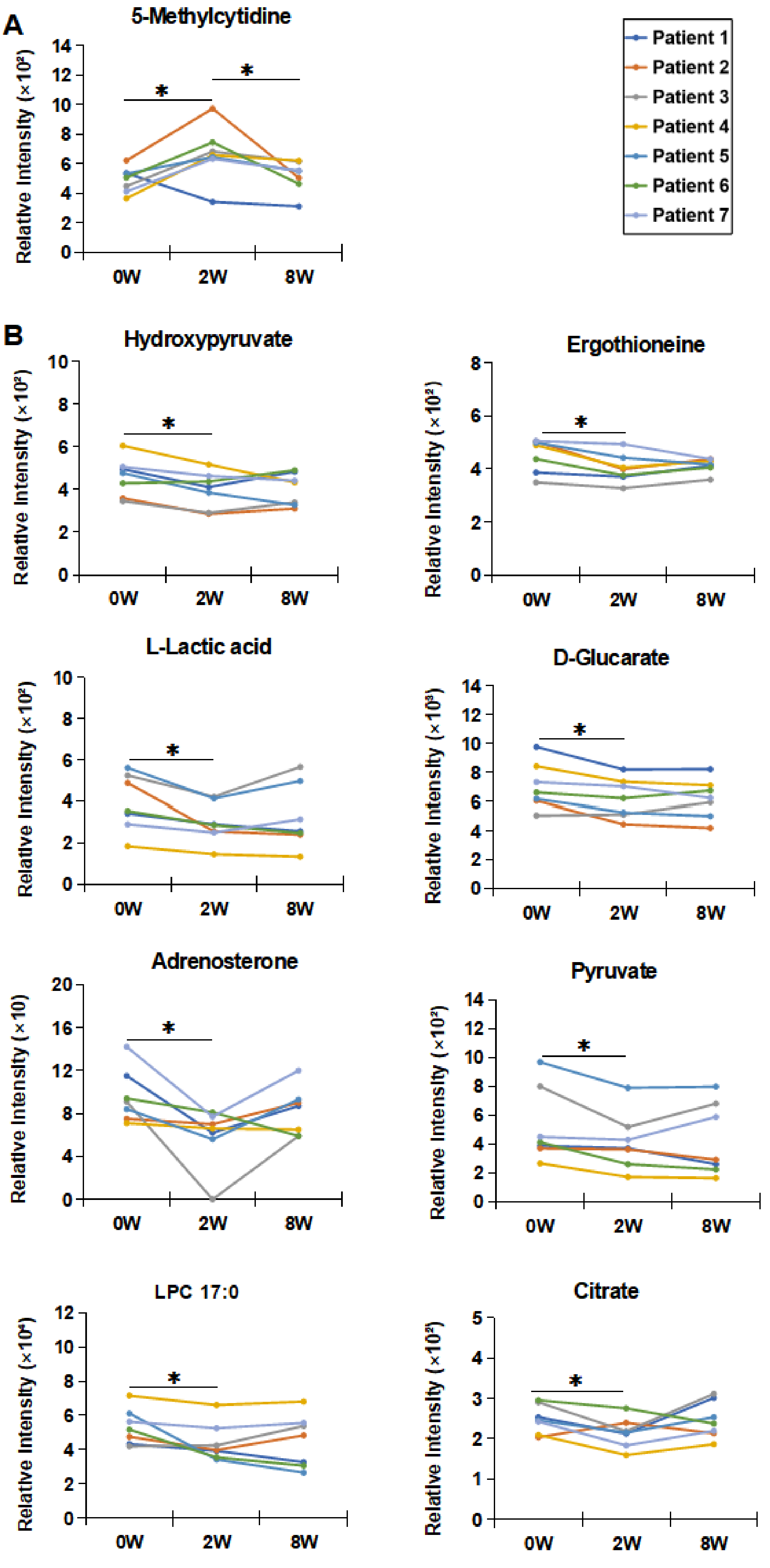

A
5-Methylcytidine
Patient 1
Patient 2
Patient 3
Patient 4
Patient 5
Patient 6
Patient 7
B
Hydroxypyruvate
Ergothioneine
L-Lactic acid
D-Glucarate
Adrenosterone
Pyruvate
LPC 17:0
Citrate
Relative Intensity
0W
2W
8W

Figure S4. Individual patient trajectories of serum metabolites markedly altered by 2W PEM treatment.

(A) Time-course profiles of a metabolite significantly increased at 2 weeks (2W). **(B)** Time-course profiles of metabolites significantly decreased at 2W. Metabolite levels were measured at 0, 2, and 8 weeks and are shown as individual patient trajectories (n = 7). Statistical significance was evaluated by paired two-tailed Student's *t*-test (**P*<0.05, ***P*<0.01). LPC, lysophosphatidylcholine.

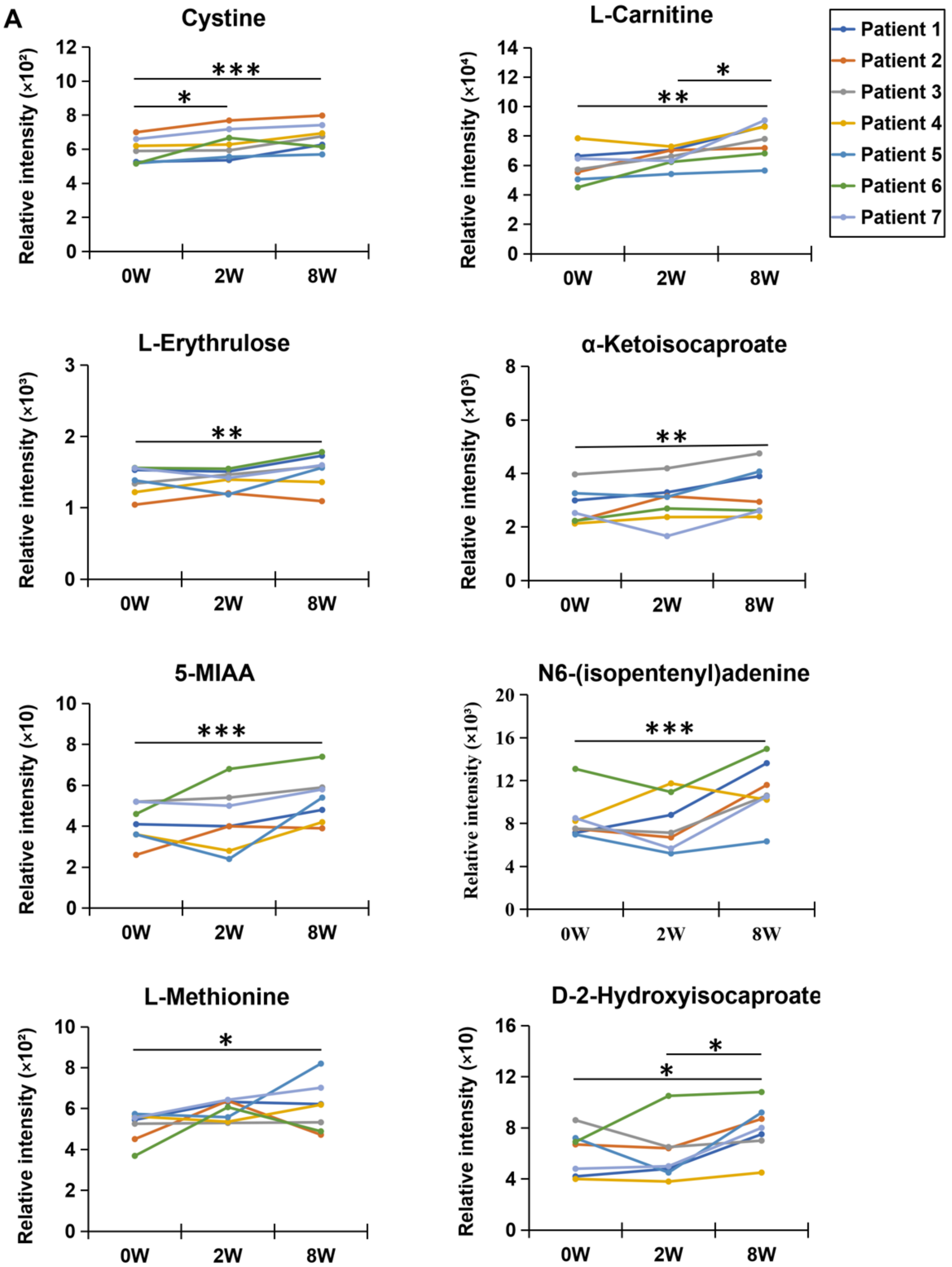

A
Cystine
L-Carnitine
Patient 1
Patient 2
Patient 3
Patient 4
Patient 5
Patient 6
Patient 7
L-Erythrulose
α-Ketoisocaproate
5-MIAA
N6-(isopentenyl)adenine
L-Methionine
D-2-Hydroxyisocaproate
Relative intensity (×10²)
Relative intensity (×10⁴)
Relative intensity (×10³)
Relative intensity (×10)
0W
2W
8W

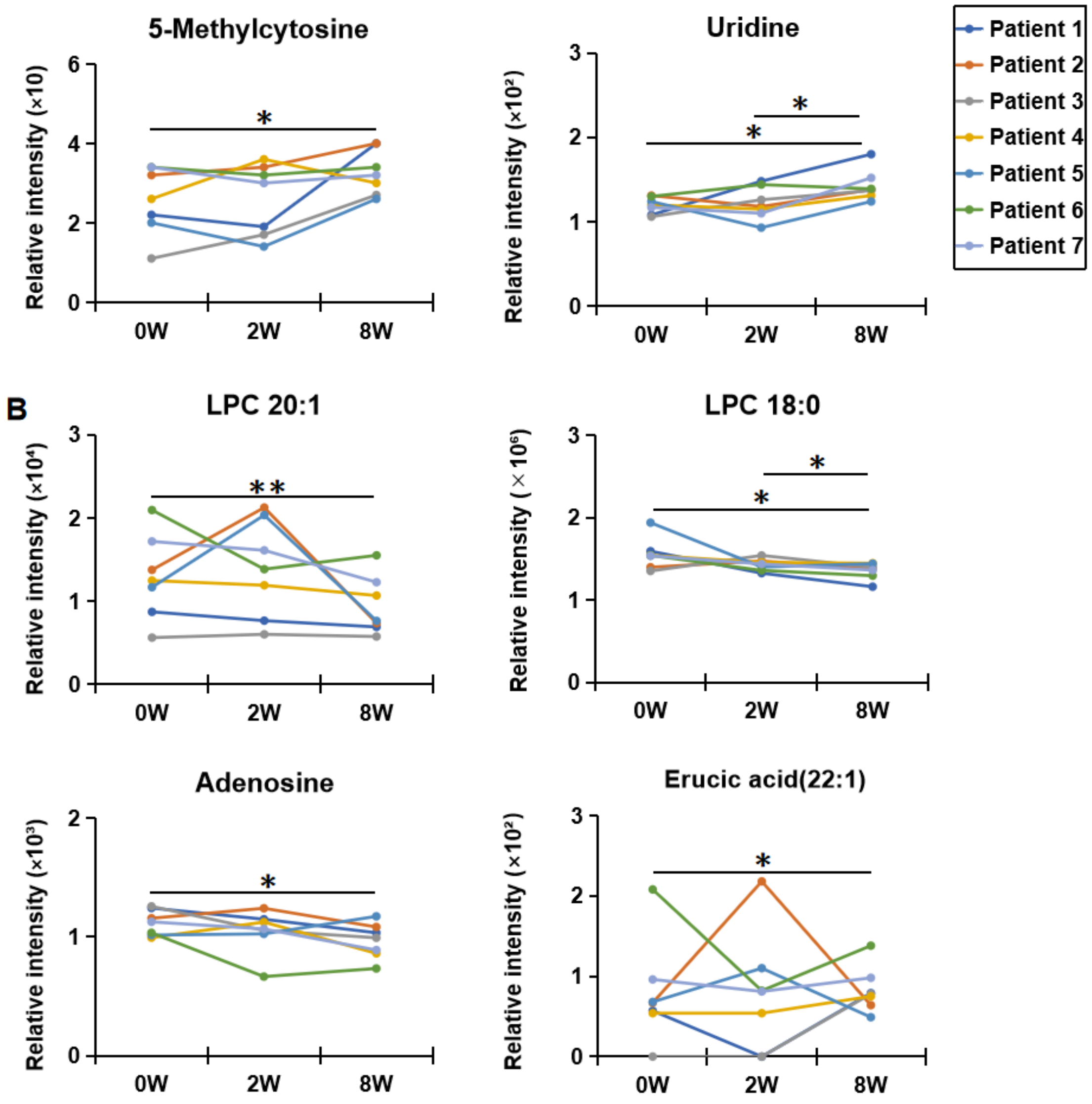

Figure S5. Individual patient trajectories of serum metabolites markedly altered by 8W PEM treatment.

(A) Time-course profiles of metabolites significantly increased at 8 weeks (8W). **(B)** Time-course profiles of metabolites significantly decreased at 8 weeks. Metabolite levels were measured at 0, 2, and 8 weeks and are shown as individual trajectories (n = 7). Statistical significance was evaluated by paired two-tailed Student's *t*-test (**P*<0.05, ***P*<0.01, ****P*<0.001). MIAA, 5-methoxyindoleacetic acid; LPC, lysophoshatidylcholine.

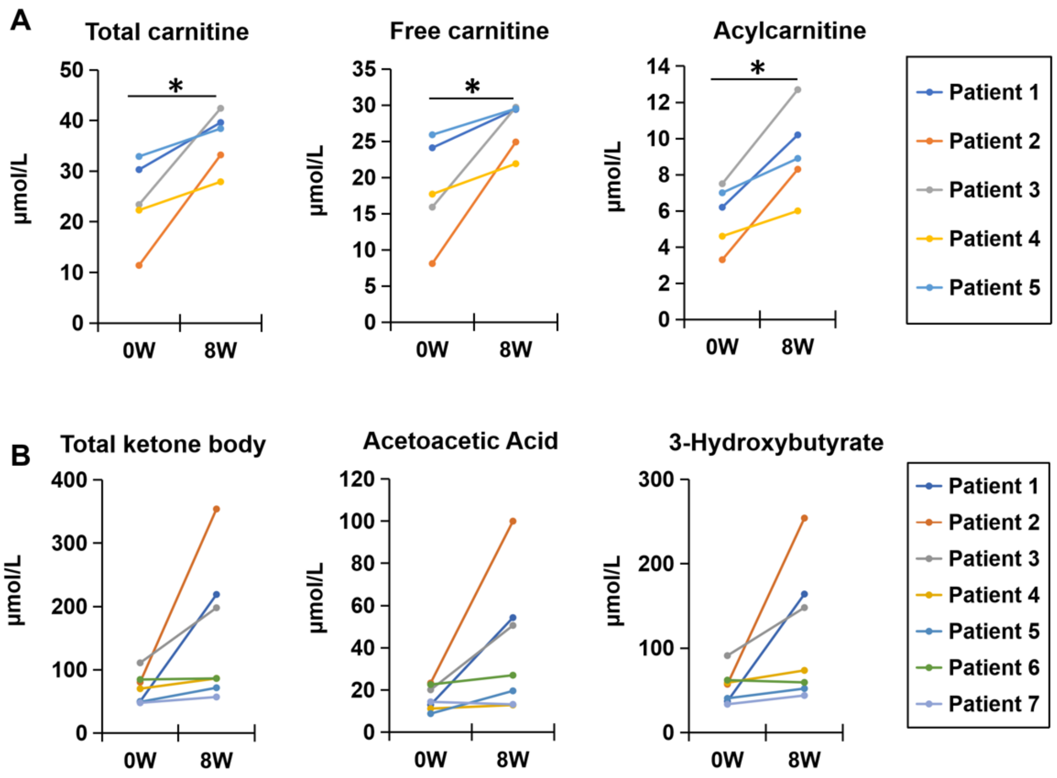

Figure S6. Individual changes in serum carnitine and ketone bodies concentrations from baseline (0W) to 8 weeks (8W) following PEM treatment.

(**A**) Serum total carnitine, free carnitine, and acylcarnitine concentrations. (**B**) Serum total ketone body, acetoacetic acid, and 3-hydroxybutyrate. Data are shown as individual patient trajectories (carnitine, n = 5; ketone bodies, n = 7). Statistical significance was evaluated by paired two-tailed Student's *t*-test (*P<0.05).

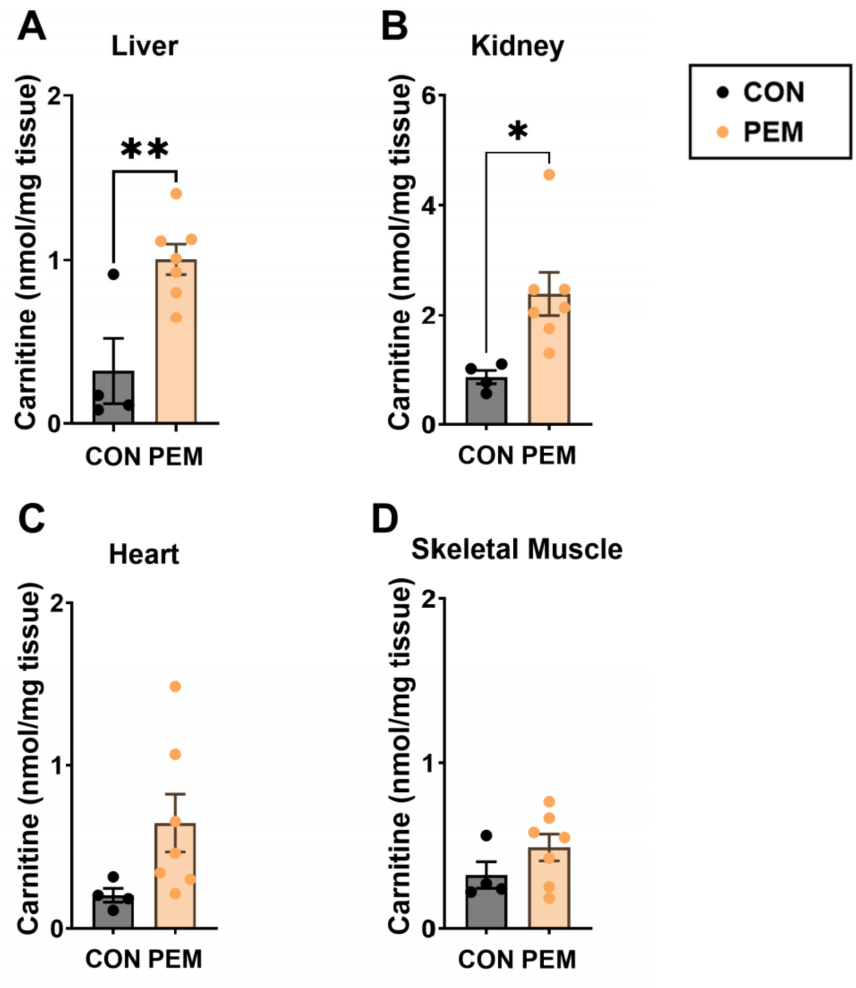

Figure S7. Carnitine concentrations (nmol/mg tissue) in mouse tissues following PEM treatment.

(**A–D**) Carnitine concentrations in liver (**A**), kidney (**B**), heart (**C**), and skeletal muscle (**D**) after 2-week PEM treatment in mice. Data are presented as mean ± SEM. The statistical significance was evaluated by unpaired two-tailed Student's *t*-test (*$P<0.05$, **$P<0.01$). CON, vehicle-treated mice; PEM, mice treated with a clinically relevant dose of PEM (0.1 mg/kg/day).

Table S1. Baseline characteristics of the study participants.

Data are presented as mean ± SEM or n (%). BMI, body mass index; MASLD, metabolic dysfunction-associated steatotic liver disease; BMI, HDL-C, high-density lipoprotein cholesterol; LDL-C, low-density lipoprotein cholesterol; AST, aspartate aminotransferase; ALT, alanine aminotransferase; ALP, alkaline phosphatase; γ-GTP, gamma-glutamyltransferase; HbA1c, hemoglobin A1c.

Characteristics	Values
Participants, n	7
Age, years	67 ± 4
Female/Male, n	7/0
Body weight, kg	57.9 ± 4.2
BMI, kg/m²	24.6 ± 1.7
MASLD, n (%)	7 (100)
Hypertriglyceridemia, n (%)	7 (100)
Hypertension, n (%)	7 (100)
Diabetes mellitus, n (%)	3 (43)
Hyperuricemia, n (%)	2 (29)
Statin use, n (%)	3 (43)
Total cholesterol, mg/dL	208 ± 12
Triglycerides, mg/dL	191 ± 22
HDL-C, mg/dL	50 ± 4
LDL-C, mg/dL	136 ± 9
AST, U/L	65 ± 11
ALT, U/L	70 ± 4
ALP, U/L	289 ± 27
γ-GTP, U/L	146 ± 54
Fasting blood glucose, mg/dL	116 ± 9
HbA1c, %	6.0 ± 0.4

Table S2. Up-regulated metabolites by 2W PEM treatment.

Top	Name	Rt	Ms	Category	0W Mean	2W Mean	Ratio (2W/0W)	*P* value (2W/0W)
1	5-Methylcytidine	0.68	256.0906	Nucleotide metabolism	490.57	669.71	1.365	0.0389
2	Cystine	10.33	241.0307	Nucleotide metabolism	590	638.29	1.082	0.0494

Rt, retention time (min); Ms, mass-to-charge ratio (m/z).

Table S3. Down-regulated metabolites by 2W PEM treatment.

Top	Name	Rt	Ms	Category	0W Mean	2W Mean	Ratio (2W/0W)	*P* value (2W/0W)
1	Hydroxypyruvate (β-Hydroxypyruvic acid)	0.66	103.0029	Glycine, Serin metabolism	460.29	399.29	0.867	0.0038
2	Ergothioneine	0.66	230.1009	Histidine metabolism	453.57	402.71	0.888	0.0092
3	L-Lactic acid	12.08	89.024	Glycolysis	39278.43	29584	0.753	0.0108
4	D-Glucarate	0.66	209.0283	Glucuronic acid metabolism	7054.29	6235.29	0.884	0.0143
5	Adrenosterone	6.92	359.1804	Steroids	96	58.86	0.613	0.0256
6	Pyruvate	13.33	87.008	Glycolysis	522.14	414.29	0.793	0.0319
7	LPC 17:0 (1-Heptadecanoyl-glycero-3-phosphocholine)	8.46	510.3576	Lyso PC	53441	44342.14	0.83	0.043
8	Citrate	1.13	191.0251	Citrate cycle (ATP cycle)	249.14	215.43	0.865	0.045

Rt, retention time (min); Ms, mass-to-charge ratio (m/z); LPC, lysophosphatidylcholine.

Table S4. Up-regulated metabolites by 8W PEM treatment.

Top	Name	Rt	Ms	Category	0W Mean	8W Mean	Ratio (8W/0W	*P* value (8W/0W)
1	Cystine	10.33	241.0307	Nucleotide metabolism	590	674.43	1.143	<0.0001
2	L-Carnitine	8.25	162.1132	Lysine metabolism	59702.71	76873.57	1.288	0.0010
3	L-Erythrulose	0.70	119.0343	Sugar	1376.71	1530.43	1.112	0.0024
4	α-Ketoisocaproate (4-Methyl-2-oxopentanoate)	11.17	129.0541	Valine, leucine and isoleucine metabolism	2759.14	3321.57	1.204	0.0036
5	5-MIAA 6-(5-Methoxyindoleacetate)	10.05	204.0681	Tryptophan metabolism	41.29	53.43	1.294	0.0083
6	6-(isopentenyl)adenine	8.72	204.1228	Plant metabolites	8426.29	11120.71	1.320	0.0181
7	L-Methionine	10.10	150.0574	Amino acid	511.71	608.14	1.188	0.0216
8	D-2-Hydroxyisocaproate	10.53	131.0721	Organic acid	60.57	79.57	1.314	0.0389
9	5-Methylcytosine	7.58	126.0586	Pyrimidine metabolism	25.57	32.71	1.279	0.0465
10	Uridine	15.57	245.0743	Pyrimidine metabolism	119.43	143	1.197	0.0465

Rt, retention time (min); Ms, mass-to-charge ratio (m/z); 5-MIAA, 5-methoxyindoleacetic acid.

Table S5. Down-regulated metabolites by 8W PEM treatment.

Top	Name	Rt	Ms	Category	0W Mean	8W Mean	Ratio (8W/0W	*P* value (8W/0W)
1	LPC 20:1 (1-Eicosenoyl-glycero-3-phosphocholine)	9.01	550.3842	LPC	12888.86	9416	0.731	0.0081
2	LPC 18:0 (1-Stearoylglycerophosphocholine)	8.84	524.3728	LPC	1555963	1353294.86	0.870	0.0390
3	Adenosine	1.08	268.1034	Purine metabolism	1117.43	966.43	0.865	0.0433
4	Erucic acid (22:1)	15.34	337.3091	Fatty Acid	78.57	49.57	0.631	0.0454

Rt, retention time (min); Ms, mass-to-charge ratio (m/z); LPC, lysophosphatidylcholine.

Table S6. Absolute tissue weights used for normalization of tissue carnitine concentrations.

Mouse ID	Liver (mg)	Kidney (mg)	Heart (mg)	Skeletal muscle (mg)
Con1	26.1	16.0	8.0	24.7
Con2	22.7	16.5	14.7	22.3
Con3	26.8	16.0	11.8	23.0
Con4	27.8	17.2	11.0	19.9
PEM1	19.0	14.0	8.2	29.3
PEM2	18.0	15.0	12.8	24.6
PEM3	27.1	20.3	9.0	31.7
PEM4	31.0	16.5	14.3	31.0
PEM5	24.1	13.0	14.7	22.6
PEM6	20.6	12.0	9.6	17.0
PEM7	29.9	12.2	8.0	28.0

Supplementary Table S7. Primer pairs used for qPCR analysis.

F, forward sequence; R, reverse sequence.

Gene	Accession #	Primer sequence (3')
18S rRNA	NR_003278	F 5‘-CACGGACAGGATTGACAGATTG-3’<br>R 5‘-CAGACAAATCGCTCCACCAA-3’
Aldh9a1	NM_001402839	F 5‘-ACGGAGAAGAAAGGATCGCTA-3’<br>R 5‘-TTTCATCCTTCCGCTCCCAA-3’
Bbox1	NM_001413162	F 5‘-TTCAAGATGAATCCAGGTGATGTG-3’<br>R 5‘-ACTAAAGTCTAAGGCCAGGAGAT-3’
Cpt1a	NM_013495	F 5‘-TGGCATCATCACTGGTGTGTT-3’<br>R 5‘-GGTCCGATTGATCTTTGCAATC-3’
Cpt1b	NM_009948.2	F 5’-AGGCACTTCTCAGCATGGTC-3’<br>R 5’-ACGGACACAGATAGCCCAGA-3’
Cpt2	NM_009949	F 5‘-ATCGTACCCACCATGCACTAC-3’<br>R 5‘-CTGTCATTCAAGAGAGGCTTCTG-3’
Crat	NM_007760	F 5‘-AGGGCCAAGAAAATGGAGAA-3’<br>R 5‘-CTCCTGGGCTGGAGTAGATG-3’
Crot	NM_023733	F 5‘-AAGCCAAAGCCCAACATCTC-3’<br>R 5‘-GAGCGAGCTGAATAAAGGTATCAG-3’
Slc22a5	NM_001362711	F 5‘-ACAGCTCTCAGACAGGTTTGGTC-3’<br>R 5‘-ACCTGATGTCCATACCCAGGAC-3’
Slc25a20	NM_020520	F 5‘-GAGCCGAAACCCATCAGTCC-3’<br>R 5‘-CAGTCGGACCTTGACCGTG-3’
Tmlhe	NM_001177307	F 5‘-TTTCAAGAGCCCTGTGGGTACA-3’<br>R 5‘-TTGTTAGGGTTCGGTGTGCTG-3’

Gene names:
Aldh9a1, aldehyde dehydrogenase 9A1; *Bbox1,* gamma-butyrobetaine hydroxylase 1; *Cpt1,* carnitine palmitoyltransferase 1a; *Cpt1b,* carnitine palmitoyltransferase 1b; *Cpt2,* carnitine palmitoyltransferase 2; *Crat,* carnitine acetyltransferase; *Crot,* carnitine O-octanoyltransferase; *Slc22a5,* solute carrier family 22 member 5; *Slc25a20,* mitochondrial carnitine/acylcarnitine translocase; *Tmlhe,* trimethyllysine dioxygenase.
